# Construction and Characterization of Emulsions Stabilized by Whey Protein Isolate-Naringin-Sodium Alginate Ternary Complex

**DOI:** 10.3390/foods15010019

**Published:** 2025-12-22

**Authors:** Si Chen, Mengmeng Wei, Guoyang Liu, Benguo Liu

**Affiliations:** School of Food Science, Henan Institute of Science and Technology, Xinxiang 453003, China; chensi@hist.edu.cn (S.C.); weimengmeng@stu.hist.edu.cn (M.W.); liuguoyang@stu.hist.edu.cn (G.L.)

**Keywords:** ternary complex, emulsion, stability, delivery system, microrheological properties

## Abstract

This study constructed ternary complexes of whey protein isolate (WPI), naringin (NAR), and sodium alginate (SA) and their stabilized emulsions, and characterized their physicochemical properties. The results confirmed interactions between NAR and SA with WPI, which induced quenching of WPI intrinsic fluorescence; formation of the ternary complexes could effectively inhibit WPI flocculation near its isoelectric point. Compared with the corresponding binary complexes, the WPI-NAR-SA ternary complexes developed more stable emulsions with smaller droplet sizes and higher absolute ζ-potential values, exhibiting superior storage stability that increased with SA concentration. After storage at 4 °C for 20 days, the emulsion system exhibited a peroxide value remaining below 4.7 μg/mL; after 9 days of UV irradiation, lutein retention reached 82.49%. Both protective effects increased with rising SA concentration. This study provides a strategy for the development of a novel functional emulsion delivery system.

## 1. Introduction

Whey protein, due to its balanced essential amino acid composition and superior nutritional value, has garnered significant attention in the food industry. It possesses excellent functional properties, including solubility, emulsification, foaming, and gelation characteristics, making it an ideal material for constructing emulsion systems [1]. During the emulsification process, whey protein can adsorb onto the oil–water interface, effectively stabilizing the emulsion by reducing interfacial tension, forming viscoelastic films, and providing electrostatic repulsion and steric hindrance [2]. However, emulsions stabilized by a single protein are quite sensitive to environmental conditions. When the pH approaches the isoelectric point of whey protein (approximately pH 5.0), the net charge of protein molecules approaches zero, weakening intermolecular electrostatic repulsion, which makes the emulsion prone to flocculation and precipitation. This significantly limits its application in acidic food systems. Naringin, a representative flavanone glycoside predominantly found in Citrus species, has garnered significant attention due to its versatile pharmacological spectrum, exhibiting superior antioxidant capacity, anti-inflammatory effects, and potential in lipid metabolism regulation [3].

To improve the emulsification stability of whey protein in acidic environments, the construction of complexes via electrostatic interactions between polysaccharides and proteins is an effective strategy. These polysaccharide-protein complexes not only reduce interfacial tension but also enhance the adsorption of polysaccharides at the interface, thereby achieving superior emulsification performance at lower concentrations [4,5]. The introduction of polysaccharides can alter the droplet surface charge, increase the thickness of the interfacial layer, and enhance electrostatic repulsion and steric hindrance between droplets. This, in turn, improves the coalescence stability and overall kinetic stability of the emulsion [6]. Zeng et al. significantly enhanced the elastic modulus of the interfacial film and the thermal centrifugation stability of emulsions at the same oil phase ratio by adding guar gum to a whey protein microparticle system [7]. Harnsilawat et al. also reported that the addition of alginate or carrageenan could effectively enhance the environmental stability of emulsions prepared with β-lactoglobulin [8]. Chen et al. found that, compared to individual bovine serum albumin or sugar beet pectin, the emulsion system formed by their complex exhibited superior emulsifying properties and was less susceptible to environmental factors [9]. However, although protein-polysaccharide binary complexes enhance emulsion stability, they often lack sufficient antioxidant capacity. In contrast, protein-polyphenol complexes exhibit strong bioactivities. The incorporation of polyphenols endows the composite system with enhanced bioactivities, and their interaction can induce protein structural changes, stabilizing the emulsion through synergistic effects. Previous studies have confirmed that the interaction between naringin and WPI could drive the migration and specific accumulation of the polyphenol at the oil–water interface. This unique interfacial localization enabled it to function as a potent interfacial antioxidant barrier, providing critical support for the development of functional emulsion carriers with superior oxidative stability [10]. Fan et al. reported that emulsions stabilized by (-)-epigallocatechin-3-gallate (EGCG)-protein complexes exhibited superior oxygen radical scavenging capacity and reducing power compared to those stabilized by whey protein alone [11]. However, the ability of polyphenols to improve emulsification and storage stability remains limited. To achieve both physical stability and functional activity, protein-polyphenol-polysaccharide ternary complex systems have attracted extensive attention in recent years. In such systems, proteins act as the primary emulsifiers by adsorbing at the interface, polysaccharides enhance steric hindrance to prevent droplet aggregation, and polyphenols impart pronounced antioxidant properties, thereby effectively retarding lipid oxidation. Liu et al. reported that the ternary complex exhibited superior thermal stability, emulsifying properties, and emulsion stability compared to single proteins or binary complexes, and provided enhanced protection for β-carotene [12]. The whey protein-rutin-chitosan ternary complex prepared by Huang et al. significantly reduced interfacial tension and formed an elastic interfacial film, resulting in emulsions with excellent storage stability [13]. Chen et al. also found that the chondroitin sulfate-quinoa protein isolate-dihydromyricetin composite nano-delivery system exhibited superior physical stability and environmental tolerance, while its antioxidant and anti-inflammatory bioactivities surpassed those of the corresponding binary systems [14].

Although previous studies have demonstrated the advantages of ternary complexes, their stabilization mechanisms under the critical pH condition of the protein isoelectric point, as well as the specific contributions of polyphenols, remain insufficiently elucidated. Therefore, this study constructed the whey protein (WPI)-naringin (NAR)-sodium alginate (SA) ternary complexes and systematically evaluated how the ternary composition influenced its formation and the properties of the resulting emulsions under isoelectric point conditions. The aim is to clarify the superiority of ternary complex systems and to provide strategic insights for developing highly stable functional emulsion delivery systems.

## 2. Materials and Methods

### 2.1. Materials and Chemicals

WPI (93.77% protein) was purchased from Mullins Whey Co. (Mosinee, WI, USA). NAR (purity ≥ 95%) and lutein were obtained from Aladdin Biochemical Technology Co., Ltd. (Shanghai, China). SA and medium-chain triglycerides (MCT) were purchased from Shanghai Yuanye Biotechnology Co., Ltd. (Shanghai, China). All other chemical reagents were of analytical grade.

### 2.2. Preparation of Ternary Complex

A mixed solution containing 0.9% WPI (*w*/*w*) and 0.04% NAR (*w*/*w*) was prepared using phosphate buffer (50 mM, pH 6.8) as the solvent. This solution was then combined in equal volumes with SA solutions of different mass fractions (0.45%, 0.90%, and 1.8%; *w*/*w*) to obtain ternary complexes, which were subsequently designated as WPI-NAR-SA-1, WPI-NAR-SA-2, and WPI-NAR-SA-3.

### 2.3. Turbidity Measurement of Ternary Complex

The pH of the composite solutions was adjusted to 4.0, 5.0, and 6.0 using 1 M HCl, and their visual appearance was observed and recorded. The turbidity of the samples was determined referring to the method described by Archut et al. [15] with slight modifications. Briefly, the turbidity was measured at 25 °C using a 2100N laboratory turbidimeter (Hach Company, Loveland, CO, USA). A 0.45% (*w*/*w*) WPI solution and a 0.45% (*w*/*w*) WPI-0.02% (*w*/*w*) NAR mixture were used as blank controls.

### 2.4. Fluorescence Spectroscopy Measurement of Ternary Complex

The fluorescence spectra of WPI, WPI-NAR, and WPI-NAR-SA at pH 5.0 were acquired using a Cary Eclipse fluorescence spectrophotometer (Agilent Technologies, Santa Clara, CA, USA), following the procedure described by Wang et al. [16] with minor modifications. Prior to measurement, the WPI concentration in all samples was maintained consistent. The samples were then equilibrated in a water bath at 37 °C for 20 min. The experimental parameters were set as follows: excitation wavelength, 280 nm; emission wavelength range, 290–450 nm; excitation and emission slit widths, 5 nm; and photomultiplier tube voltage, 700 V.

### 2.5. Preparation of Emulsion

The WPI-NAR and WPI solutions were separately mixed with MCT at a 9:1 oil-to-water ratio. The mixtures were pre-homogenized using an Ultra-Turrax T18 high-speed dispersion (IKA, Staufen, Germany) at 15,000 rpm for 3 min to form coarse emulsions. These coarse emulsions were then ultrasonicated using a cell disruptor (JY99-IIDN, Scientz, Ningbo, China) at 400 W for 180 s in an ice bath, yielding the primary emulsions PE-1 and PE-2. Primary emulsion PE-1 was combined with an equal volume of SA solution at varying concentrations (0.45%, 0.9%, and 1.8% (*w*/*w*)). After magnetic stirring for 2 h and pH adjustment to 5.0, the resulting secondary emulsions were designated SE-1, SE-2, and SE-3. Similarly, PE-2 was mixed with an equal volume of 0.9% (*w*/*w*) SA solution, stirred for 2 h, adjusted to pH 5.0, and labeled SE-4. To clarify the individual roles of NAR and SA, control emulsions (control-1, control-2, control-3) were prepared by replacing these components with equivalent amounts of WPI under identical processing conditions. The detailed composition of each emulsion is provided in Table 1. Sodium azide was added to all emulsions as a microbial inhibitor.

### 2.6. Determination of Emulsion Oil Droplet Size and ζ-Potential

The droplet size of the emulsion was determined using a laser particle size analyzer (Model BT-9300H, Bettersize Instruments Ltd., Dandong, China), and the ζ-potential was measured using a potential/particle size/molecular weight analyzer (Model BT-Zeta100, Bettersize Instruments Ltd., Dandong, Liaoning, China). All measurements were conducted at 25 °C. To minimize deviations caused by multiple light scattering, the emulsions were diluted 1000-fold with a buffer of identical pH to that of the sample prior to measurement.

### 2.7. Optical Microscopy Observation

A 15 μL aliquot of the emulsion was placed on a glass slide and covered with a coverslip to prevent bubble formation. The sample morphology was observed under a BH200P polarizing microscope (Sunny Hengping Scientific Instruments Co., Ltd., Shanghai, China) at 200× magnification, and images were captured for documentation and analysis.

### 2.8. Micro-Rheological Characterization of Emulsions

The microrheological properties of the emulsion samples were measured using a Rheolaser LAB 6 microrheometer (Formulaction, Toulouse, France). The freshly prepared emulsions were promptly transferred into cylindrical test tubes and monitored for 3 h at 25 °C using a charge-coupled detector (CCD). Data were recorded and analyzed with Rheosoft Master 1.4 software.

### 2.9. Determination of Lipid Oxidation

The determination was carried out according to the method of He et al. [17] with slight modifications. The extent of lipid oxidation in emulsions stored at different temperatures (4 °C and 30 °C) was evaluated by determining the content of primary oxidation products-lipid hydroperoxides. An aliquot of 0.2 mL emulsion was mixed with 1.5 mL of an iso-octane/isopropanol solution (3:1, *v*/*v*) and vortexed for 1 min. The mixture was then centrifuged at 3000 rpm for 6 min at 25 °C, after which 200 μL of the supernatant was added to 2.8 mL of a methanol/1-butanol solution (2:1, *v*/*v*). Subsequently, 15 μL of 3.94 mol/L ammonium thiocyanate and 15 μL of a ferrous solution (prepared by mixing 0.132 mol/L barium chloride and 0.144 mol/L ferrous sulfate) were added. The mixture was vortexed for 30 s and left to stand at room temperature for 20 min. The absorbance was then measured at 510 nm using a TU-1810 UV-Vis spectrophotometer (Beijing Purkinje General Instrument Co., Ltd., Beijing, China). The hydroperoxide content in the emulsion was calculated based on a hydrogen peroxide standard calibration curve.

### 2.10. Determination of Lutein Stability

Following the experimental method described by Wang et al. [18], lutein was dissolved in MCT to prepare an oil phase with a lutein concentration of 1.0 mg/mL. Lutein-loaded emulsions (b-SE-1 to b-SE-4) were then prepared according to the procedure outlined in Section 2.6. The emulsions were placed 20 cm below a 6 W ultraviolet lamp and maintained at 30 °C to monitor the degradation trend of lutein. At predetermined intervals, 1 mL of the lutein emulsion was sampled and mixed with 6 mL of a hexane/ethanol solution (2:1, *v*/*v*) and 1 mL of distilled water. The mixture was vortexed for 1 min and centrifuged (2000× *g*, 6 min) using a high-speed centrifuge. After standing in the dark for 10 min, the absorbance of the supernatant was measured at 450 nm using a UV-Vis spectrophotometer. An MCT sample with the same lutein concentration served as the experimental control. The lutein content was calculated according to the standard calibration curve, and the lutein retention rate was determined using the following formula:

$$\mathrm{Retention}\;\mathrm{rate}=\;\frac{C_{t}}{C_{0}}\times 100\%$$

In the equation, *C*_0_ represents the initial lutein content in the sample, while *C_t_* denotes the lutein content in the sample after exposure to ultraviolet light for a duration of t.

### 2.11. Statistical Analysis

All experiments were performed in triplicate, and the results were expressed as mean ± standard deviation. Significant differences among treatments were analyzed using the IBM SPSS Statistics 21.0 software with Duncan’s multiple range test (*p* < 0.05). Graph plotting was conducted using Origin 2018 software.

## 3. Results and Discussion

To systematically elucidate the stabilizing mechanism and functional potential of the ternary system, this study employed a multi-stage experimental design. Initially, the intermolecular interactions within the WPI-NAR-SA complexes were investigated to confirm their formation mechanism (Section 3.1 and Section 3.2). Subsequently, these complexes were applied to stabilize emulsions near the protein’s isoelectric point (Section 3.3, Section 3.4 and Section 3.5). Finally, the functional performance of the system regarding lipid oxidation and lutein protection was evaluated (Section 3.6 and Section 3.7). To clarify the specific contribution of each component within the system, distinct control groups were designed, as detailed in Table 1. By comparing emulsions SE-1 through SE-3, the influence of SA concentration on emulsion properties was evaluated. SE-4, representing the NAR-free WPI-SA binary system, was utilized to isolate and assess the specific role of naringin within the ternary complex. Furthermore, Controls 1–3 were prepared by substituting the non-protein components (NAR and SA) with equivalent amounts of WPI. This design ensured that the total solid content remained constant across comparisons, confirming that the observed stability improvements were attributed to specific molecular interactions rather than merely to an increase in continuous phase viscosity or mass concentration.

### 3.1. Turbidity of Ternary Complex

**Figure 1:**
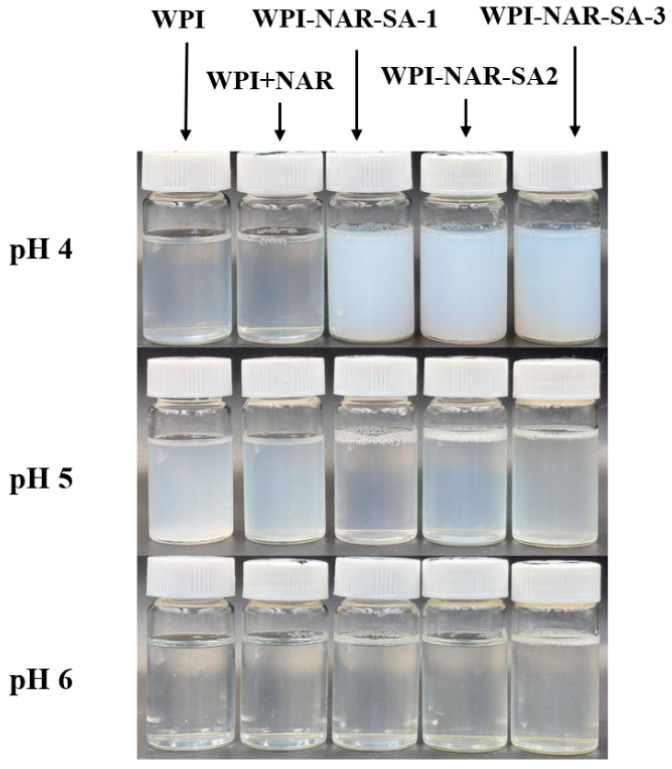
Appearance of WPI, WPI-NAR, and WPI-NAR-SA complexes at different pH values.

The pH of the system played a critical role in regulating the formation and dissociation of the complexes by modulating their surface charge density, with changes in turbidity serving as a visual indicator of their phase behavior [19,20]. The turbidity values and visual appearance of WPI, WPI-NAR, and WPI-NAR-SA complex solutions under different pH conditions are presented in Table 2 and Figure 1. At pH 4.0, both WPI and WPI-NAR exhibited slight turbidity, whereas the WPI-NAR-SA complex demonstrated a significantly higher turbidity value. This increase was attributed to the addition of SA, which induced intense electrostatic complex coacervation, leading to macroscopic phase separation [21]. At pH 5.0, precipitation occurred in WPI due to its proximity to the isoelectric point, and significant turbidity was also observed in the WPI-NAR complex. However, upon incorporation of SA, the complex solution became clear, accompanied by a marked decrease in turbidity. This clearing phenomenon can be explained by the adsorption of SA onto the protein surface, which imparted a strong negative charge. The resulting electrostatic repulsion stabilized the proteins against aggregation near the isoelectric point, thereby preventing phase separation and facilitating the formation of stable soluble complexes. At pH 6.0, WPI, being distant from its isoelectric point, formed a clear and transparent solution, and the WPI-NAR solution also remained non-turbid. Furthermore, both WPI and SA molecules carried negative charges and remained soluble, resulting in a low turbidity value for the ternary complex system at this pH. These observations aligned with the findings of Lan et al., who reported that soluble complexes formed from pea protein and high methoxyl pectin could enhance the stability of the protein at its isoelectric point [22].

### 3.2. Fluorescence Spectra of Ternary Complex

**Figure 2:**
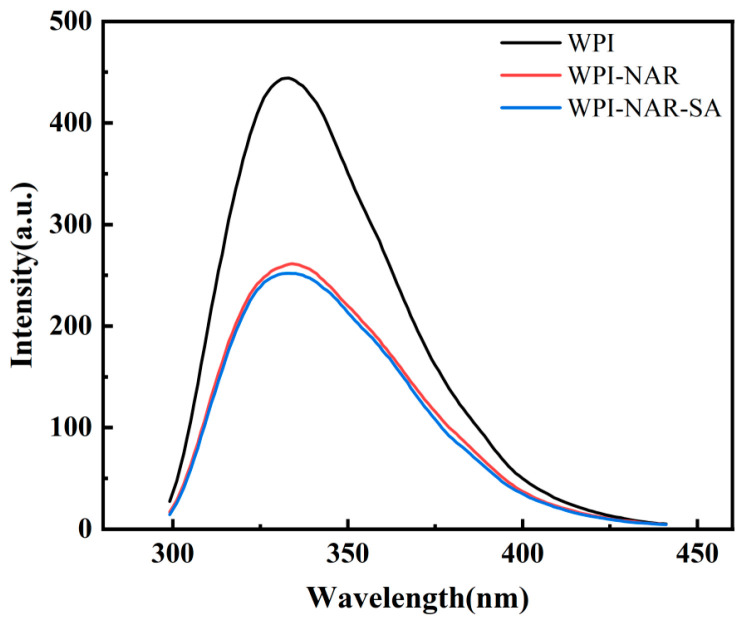
Fluorescence spectra of WPI, WPI-NAR, and WPI-NAR-SA measured at pH 5.0.

The intrinsic fluorescence of proteins was widely used to analyze the binding characteristics between proteins and small molecules. This approach was employed since the interaction between proteins and small molecules could induce alterations in the microenvironments of hydrophobic amino acids, consequently leading to shifts or quenching of the fluorescence peak [23]. Fluorescence spectra of WPI, WPI-NAR, and WPI-NAR-SA, measured at pH 5.0, are presented in Figure 2. As shown, the maximum emission wavelengths for all three sample groups were found to be consistent, with no significant shift observed. This indicated that the incorporation of NAR and SA did not markedly alter the polarity of the microenvironment surrounding the Tryptophan (Trp) residues. However, a significant reduction in fluorescence intensity from 444 a.u. for WPI alone to 261 a.u. upon the addition of NAR was recorded, suggesting that an interaction between NAR and WPI had occurred, resulting in fluorescence quenching. Following the introduction of SA, the fluorescence intensity was further quenched to 251 a.u. Given that the concentration of WPI was maintained constant throughout the experiments, the observed changes in fluorescence intensity were attributed to the sequential addition of NAR and SA. In conclusion, although the polarity of the Trp microenvironment remained unchanged, the progressive quenching of fluorescence intensity demonstrated that interactions between WPI, NAR, and SA had taken place, inducing conformational adjustments in the protein and confirming the formation of the ternary complex.

### 3.3. Droplet Size and ζ-Potential of Emulsion

**Figure 3:**
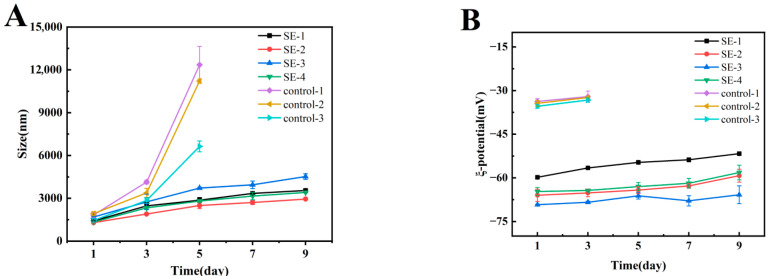
Variations in droplet size and ζ-potential of emulsion samples during storage.

The stability of emulsions was influenced by a combination of factors, among which droplet size and ζ-potential were recognized as two critical parameters. Emulsions with smaller droplet sizes generally demonstrated higher stability, as smaller oil droplets could be distributed more uniformly within the continuous phase, exhibiting more intense Brownian motion which consequently reduced the tendency for droplet aggregation [24]. The particle sizes of the emulsion samples were displayed in Figure 3A. Phase separation was observed in the control group upon storage to day 3, and the mean droplet size was found to continuously increase, which was indicative of its poor physical stability. In contrast, a slower rate of droplet size increase was recorded in all composite emulsion systems with prolonged storage time. Among all composite emulsions, SE-3 was characterized by the largest droplet size. This phenomenon could be attributed to the higher SA concentration, which tended to form larger electrostatic complexes with whey protein, ultimately resulting in larger droplet sizes. The larger droplet size of SE-1 compared to SE-2 suggested that the lower SA concentration was insufficient to form a stable composite interfacial film with whey protein, leading to increased droplet aggregation and ultimately the formation of larger droplets. Through comparison between SE-2 and SE-4, it was observed that the incorporation of NAR led to a further reduction in droplet size. This effect could be attributed to its ability to induce conformational changes in proteins or enhance interfacial repulsive forces, thereby promoting a more compact arrangement of the adsorption layer and effectively suppressing droplet aggregation.

The ζ-potential is often employed as an indicator for evaluating the physical stability of emulsions, as it reflects, to a certain extent, the strength of interactions between emulsion droplets. An increase in the absolute value of the ζ-potential corresponds to enhanced electrostatic repulsion between particles, thereby improving the physical stability of the emulsion [25]. As illustrated in Figure 3B, the control group exhibited a relatively low absolute ζ-potential value. Phase separation was observed in this group on day 3, after which no further potential measurements were conducted. In contrast, the ζ-potential values of the SE1–4 groups remained relatively stable throughout the 9-day storage period. The recorded order of absolute ζ-potential values was as follows: SE-3 > SE-2 > SE-4 > SE-1. This trend suggested that a higher proportion of SA contributed to enhanced electrostatic stability within the complex systems. The significantly lower absolute ζ-potential of SE-1 indicated that the low SA concentration was insufficient to form adequately stable complex structures with whey protein, resulting in reduced surface charge density and consequently inferior emulsion stability [26]. Under conditions of identical SA concentration, the higher absolute ζ-potential observed in the ternary complex (SE-2) compared to the binary complex (SE-4) demonstrated that the incorporation of NAR participated in the construction of the interfacial complex. This involvement led to a further increase in the charge density of the interfacial layer, thereby producing a synergistic stabilization effect, which was also consistent with the droplet size results.

### 3.4. Appearance and Microscopic Observation of Emulsion

**Figure 4:**
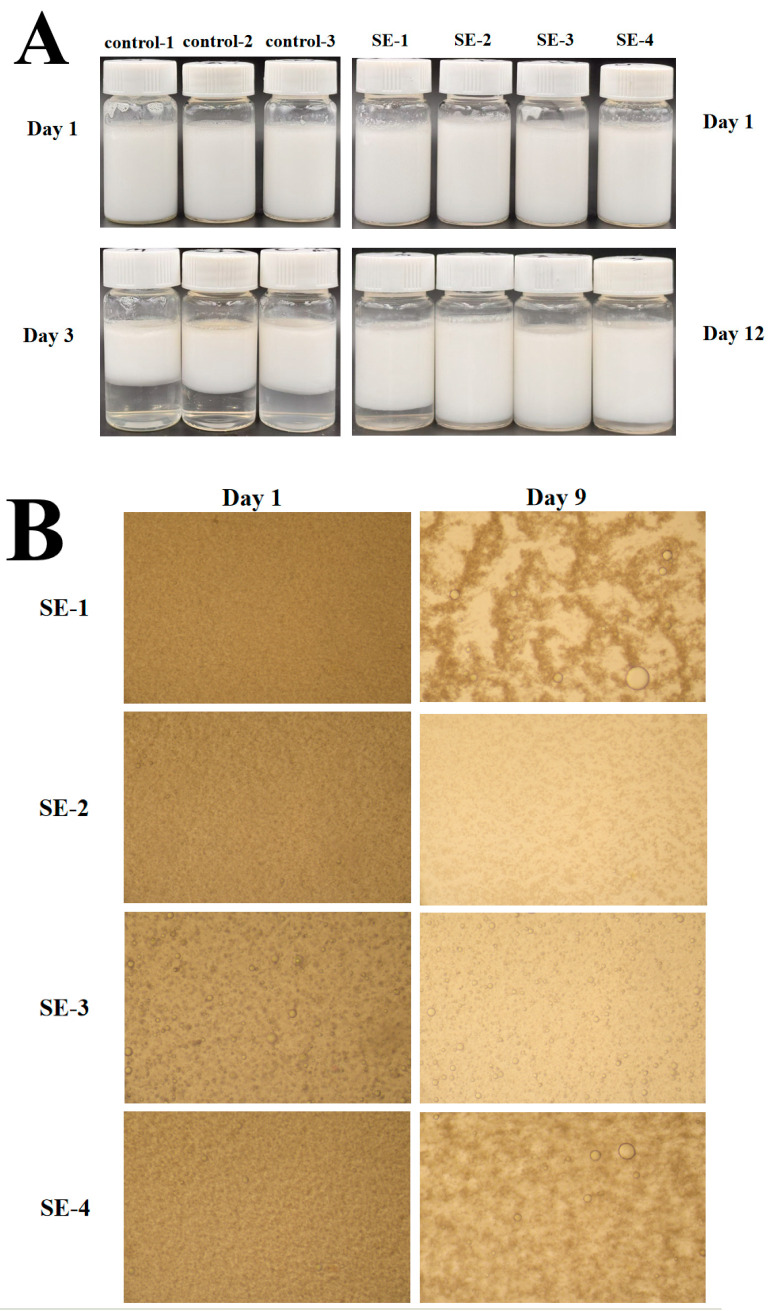
Effects of storage time on the appearance of the emulsion (**A**) and on its microscopic morphology (**B**). Note: The images presented are representative photographs selected based on visual assessment. Including images for all experimental replicates would exceed the reasonable scope of this manuscript.

The influence of storage duration on the emulsion appearance is presented in Figure 4A. Phase separation was observed in the control group on day 3 of storage, while distinct stratification was detected in SE-1 and SE-4 by day 12. Further microscopic examination of the microstructure on days 1 and 9 (Figure 4B) revealed non-uniform droplet morphology with the presence of larger droplets in SE-1 and SE-4 on day 9, indicating inferior storage stability of emulsions prepared with low-SA-concentration ternary complexes and WPI-SA binary complexes. In contrast, SE-2 maintained homogeneous droplet distribution without detectable large droplets on day 9, demonstrating uniform dispersion of the oil phase and stable emulsion structure. SE-3, containing higher SA concentration, was characterized by the formation of larger molecular aggregates, as evidenced by the presence of moderately enlarged droplets observed as early as day 1. However, the uniform droplet distribution pattern remained consistent through day 9 without significant morphological changes, confirming satisfactory storage stability of this system. These microscopic observations were consistent with previous droplet size and ζ-potential measurements, confirming that the ternary complexes exhibited superior storage stability compared to binary complexes at appropriate SA concentrations. This conclusion was also in agreement with the trend reported by Duan et al., where emulsions stabilized by whey protein isolate-sodium alginate-seaweed polyphenol ternary complexes showed no extensive aggregation after 6-day storage, while distinct droplet aggregation was observed in systems stabilized solely by protein-polyphenol binary complexes [27].

### 3.5. Microrheological Properties of Emulsion

**Figure 5:**
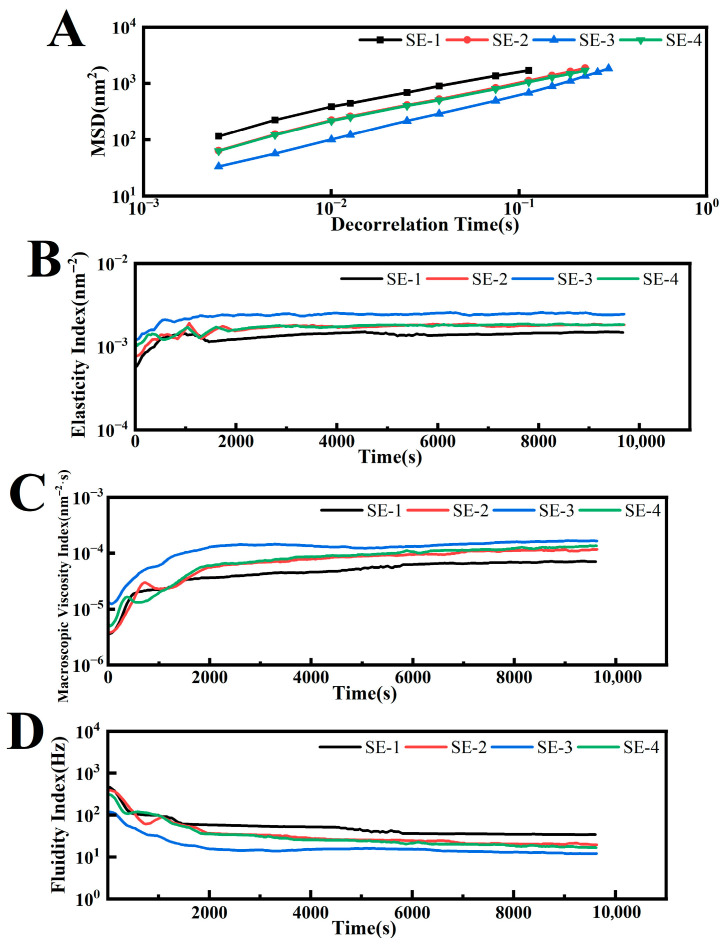
The MSD, EI, MVI, and FI curves of the emulsion samples (MSD: mean square displacement; EI: elasticity index; MVI: macroscopic viscosity Index; FI: fluidization index).

Compared with traditional mechanical rheological testing, optical microrheology enables in situ measurements of static samples without altering their shape, allowing real-time monitoring of the specimen. It has now emerged as an important technique in rheological research [28]. This method analyzes the backscattered speckle images of laser light of a given wavelength passing through the sample to obtain the motion of dispersed particles, that is, the relationship between the mean square displacement (MSD) and the decorrelation time (t), thereby characterizing the microscopic rheological properties of the dispersion. The microrheological parameters of each emulsion group at the endpoint are summarized in Table 3. The solid–liquid balance (SLB) index was calculated from the slope of the MSD curve plateau, where a steeper slope corresponded to a higher SLB index, indicating more pronounced liquid-like characteristics of particles in the system. All samples exhibited SLB values exceeding 0.5, confirming their liquid state. As shown in Figure 5A, comparison among SE-1 to SE-3 revealed that SE-3 displayed the lowest MSD values within the decorrelation time, demonstrating that increased SA concentration contributed to enhanced viscoelastic properties of the emulsion.

The elasticity index (EI) was calculated as the reciprocal of the plateau height in the MSD curves, where higher elasticity corresponded to greater EI values. As demonstrated in Figure 5B, SE-1 exhibited the lowest EI value (1.51 × 10^−3^ nm^−2^), followed by SE-2 (1.84 × 10^−3^ nm^−2^), while SE-3 displayed the highest EI value (2.42 × 10^−3^ nm^−2^). Furthermore, SE-3 achieved elastic equilibrium most rapidly among all samples. This indicated that increased SA concentration not only enhanced the elastic strength of the emulsion but also shortened the time required to reach elastic stability. The macroscopic viscosity Index (MVI) was derived from the slope of the terminal portion of the MSD curve, providing a quantitative representation of the emulsion’s macroscopic viscosity. Higher viscosity values corresponded to greater MVI values. As shown in Figure 5C, SE-3 demonstrated the highest MVI value (1.73 × 10^−4^ nm^−2^·s) at equilibrium, while SE-2 and SE-4 showed comparable values, and SE-1 registered the lowest (7.47 × 10^−5^ nm^−2^·s). These results confirmed that SE-3 possessed the highest viscosity, whereas SE-1 exhibited the lowest viscosity. The higher EI and MVI values were associated with slower particle motion, which reduced droplet collision frequency and thereby contributed to enhanced physical stability [29].

The fluidization index (FI) factor was calculated based on the characteristic time (τ) of droplet motion, reflecting the fluidity of the emulsion. During initial testing, the droplet packing structure was disrupted, while prolonged testing enabled gradual reformation of the network structure, resulting in decreasing fluidity curves that eventually reached an FI plateau. Greater fluidity in emulsions corresponded to lower viscosity. Figure 5D revealed that SE-1 displayed the highest fluidity, whereas SE-3 showed the lowest. These findings demonstrated that SA concentration significantly influenced emulsion fluidity, with elevated concentrations effectively reducing fluidity and enhancing viscosity.

### 3.6. Oxidative Stability of Emulsion Lipids

**Figure 6:**
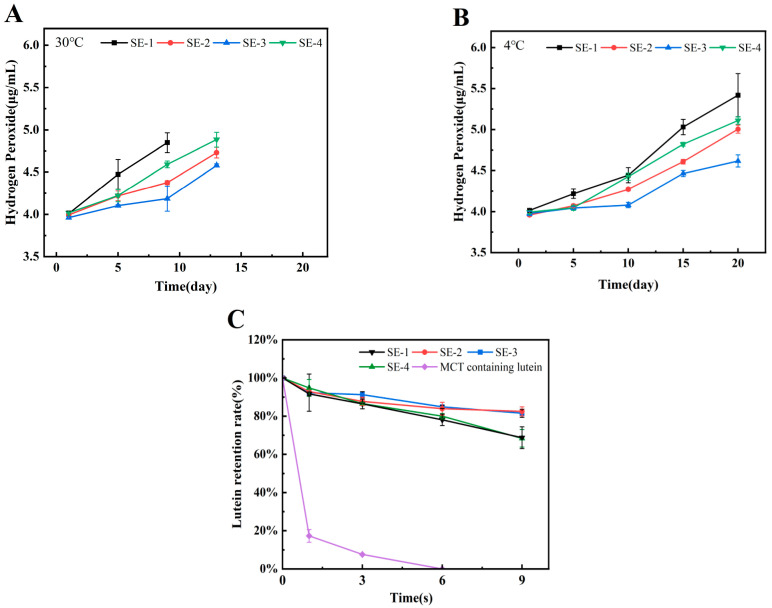
The oxidative stability ((**A**) at 30 °C; (**B**) at 4 °C) and lutein retention capacity (**C**) of the emulsion samples. Note: Monitoring was terminated when visible physical separation occurred, as samples extracted after this point could no longer represent the homogeneous oxidative state of the entire emulsion system. Consequently, the data collection ended at day 13 for samples stored at 30 °C and at day 20 for samples stored at 4 °C.

Figure 6A and Figure 6B present the hydroperoxide content variations in emulsions stored at 30 °C and 4 °C, respectively. During the initial stage, all emulsions were characterized by relatively low lipid oxidation levels. The oxidation rate was observed to be lower at 4 °C compared to 30 °C. Overall analysis revealed that regardless of storage temperature, the hydroperoxide accumulation rate followed the order of SE-1 > SE-4 > SE-2 > SE-3, indicating that increased SA concentration contributed to delayed lipid oxidation in emulsions. Comparison between SE-2 and SE-4 demonstrated that emulsions stabilized by ternary complexes possessed superior oxidative stability compared to those stabilized by conventional protein/polysaccharide binary complexes. This phenomenon was attributed to two primary mechanisms: firstly, NAR, as an effective natural antioxidant, was capable of directly scavenging lipid radicals through hydrogen atom donation, thereby exerting chemical inhibition; secondly, as indicated by previous droplet size and ζ-potential measurements, the incorporation of NAR was found to facilitate the formation of a more compact and stable WPI-NAR-SA composite interfacial membrane, which served as a physical barrier that effectively impeded contact between lipids and atmospheric oxygen [30]. Similarly, Zhou et al. reported that zein-curcumin-carrageenan (or gum arabic) ternary complex emulsions exhibited lower hydroperoxide values than zein-carrageenan (gum arabic) binary complex emulsions, further confirming the advantages of ternary complex systems in enhancing the oxidative stability of emulsions [31].

### 3.7. Lutein Protection Ability of Emulsion

Lutein is an oxygen-containing carotenoid. As the primary pigment in edible marigold flowers, lutein can filter blue light and deactivate reactive oxygen species, thereby playing an important protective role in maintaining ocular health [32,33]. However, lutein is sensitive to ultraviolet light, oxygen, and temperature, exhibiting poor stability and being prone to degradation and inactivation. This investigation evaluated the protective efficacy of emulsions prepared with ternary complexes against UV-induced lutein degradation. As shown in Figure 6C, the control group containing only lutein in MCT exhibited a sharp decrease in lutein content by day 2, with subsequent retention rates maintained below 8%. In contrast, all emulsion systems demonstrated significantly higher retention rates after 9 days of UV exposure. Specifically, SE-3 and SE-2 showed comparable lutein retention rates exceeding 80% on day 9, while SE-1 and SE-4 exhibited approximately 68% retention. Comparative analysis of SE-1 through SE-3 indicated that increased SA concentration contributed to enhanced lutein retention. Furthermore, comparison between SE-2 and SE-4 revealed that the ternary complex emulsion provided superior protection for lutein compared to the binary complex emulsion. This enhanced protection was attributed to two factors: the participation of NAR facilitated the formation of a more compact interfacial membrane, and NAR itself possessed inherent antioxidant capacity and UV-absorbing activity, thereby providing additional protection for lutein. This finding was consistent with the report by Jiang et al., who had demonstrated that β-carotene retention in emulsions prepared with whey protein-dextran-resveratrol ternary complexes was superior to those prepared with whey protein-dextran binary complexes under both UV irradiation and dark storage at different temperatures [34].

## 4. Conclusions

This study successfully developed a novel ternary complex strategy (WPI-NAR-SA) to overcome the instability of whey protein isolate (WPI) near its isoelectric point. By introducing sodium alginate (SA) into the WPI-naringin (NAR) binary system, we demonstrated that the ternary complexes formed at pH 5.0 effectively inhibited protein flocculation through enhanced electrostatic repulsion and steric hindrance. Physicochemical analyses revealed that these intermolecular interactions significantly improved the stability of the resultant emulsions, with the stabilizing effect being positively correlated with SA concentration. Furthermore, the emulsion system showed a strong capacity to delay lipid oxidation and protect lutein from degradation: after 20 days of storage at 4 °C, the peroxide value remained below 4.7 μg/mL, and after 9 days of ultraviolet irradiation, the lutein retention rate reached 82.49%. Both protective effects increased with higher SA concentrations. These findings provide a fundamental reference for the future development of environmentally stable functional delivery systems. However, there are certain limitations to this study. The current results are based on a laboratory-scale model system, which may not fully capture the complex interactions present in real food matrices. Additionally, the bioavailability of the encapsulated lutein was not evaluated. Future research should focus on the gastrointestinal digestion behavior of these ternary complex emulsions and their applications in real food products to fully validate their functional potential.

## Figures and Tables

**Table 1:** The final composition (*w*/*w*, %) of secondary emulsions and control samples.

	SE-1	SE-2	SE-3	SE-4	Control-1	Control-2	Control-3
WPI (Whey Protein Isolate, %)	0.45	0.45	0.45	0.45	0.695	0.92	1.37
SA (Sodium Alginate, %)	0.225	0.45	0.9	0.45	0	0	0
NAR (Naringin, %)	0.02	0.02	0.02	0	0	0	0
Sodium azide (%)	0.005						
MCT (Medium Chain Triglycerides, %)	5						

**Table 2:** Turbidity values (NTU) of WPI, WPI-NAR, and WPI-NAR-SA complexes under different pH conditions.

pH Treatment	WPI	WPI-NAR	WPI-NAR-SA-1	WPI-NAR-SA-2	WPI-NAR-SA-3
pH 4	57.2 ^d^ ± 0.53	51.3 ^d^ ± 2.01	1298.67 ^a^ ± 8.96	982.67 ^b^ ± 17.10	734.67 ^c^ ± 7.10
pH 5	299 ^a^ ± 1.00	193 ^b^ ± 3.46	32.57 ^e^ ± 0.32	92.6 ^c^ ± 1.35	86.77 ^d^ ±0.9
pH 6	11.67 ^d^ ± 0.29	11.6 ^d^ ± 0.20	12.87 ^c^ ± 0.32	14.23 ^b^ ± 0.21	41.87 ^a^ ± 0.67

Note: WPI-NAR-SA-1, -2, and -3 denote ternary complexes formed by mixing equal volumes of WPI-NAR solution (0.9% WPI, 0.04% NAR) with 0.45%, 0.9%, and 1.8% (*w*/*w*) SA solutions, respectively; different lowercase letters indicate significant differences between samples within the same pH group, *p* < 0.05.

**Table 3:** Representative microrheological parameters corresponding to the MSD curve in Figure 5A at 3 h.

Parameter	SE-1	SE-2	SE-3	SE-4
**SLB (solid–liquid balance)**	0.68	0.72	0.81	0.71
**EI (elasticity index; nm^−2^)**	1.51 × 10^−3^	1.84 × 10^−3^	2.42 × 10^−3^	1.82 × 10^−3^
**MVI(macroscopic viscosity Index; nm^−2^·s)**	7.47 × 10^−5^	1.22 × 10^−4^	1.73 × 10^−4^	1.43 × 10^−4^

## Data Availability

The original contributions presented in this study are included in the article. Further inquiries can be directed to the corresponding author.
